# The influence of self-compassion on mental health of postgraduates: Mediating role of help-seeking behavior

**DOI:** 10.3389/fpsyg.2022.915190

**Published:** 2022-09-16

**Authors:** Lin Min, Ni Jianchao, Lin Mengyuan

**Affiliations:** ^1^Institute of Education, Xiamen University, Xiamen, China; ^2^School of Aerospace Engineering, Xiamen University, Xiamen, China; ^3^Experimental Primary School of Xiamen, Xiamen, China

**Keywords:** postgraduates, self-compassion, help-seeking behavior, DFM, flourishing

## Abstract

This study explores the relationship between self-compassion and mental health of postgraduates based on the perspective of the dual-factor model (DFM) of mental health and the mediating role of help-seeking behavior. A total of 605 postgraduates were investigated with a questionnaire. The results showed that the DFM of mental health was better than the one-factor model for the mental health status of postgraduates. Among them, those with complete mental health accounted for the highest proportion (63.3%), followed by vulnerable (25.1%), troubled (9.1%), and symptomatic but content (2.5%). Self-compassion and non-professional help-seeking behavior had a positive predictive effect on positive mental health factors of the postgraduates, while self-compassion had a negative predictive effect on their psychological symptoms. Non-professional help-seeking behavior played a partial mediating role between self-compassion and positive mental health factors.

## Introduction

With the continuous expansion of postgraduate enrollment in China, the competition for academics and employment has become increasingly fierce. The group of postgraduates not only carries high expectations from family, society, and country but also faces multiple pressures such as uncertainty of scientific research, economic distress, marriage, employment, and interpersonal relationships. Some postgraduates even have to take on multiple responsibilities of parenthood. As a result, this group generally feels stressed and physically and mentally exhausted (Song et al., 2019). The existing school mental healthcare system pays more attention to symptom relief and crisis intervention of psychological problems. This working mode may ignore some people who need help (Wang and Zhang, 2011). Positive psychology holds that “mental health is not simply the elimination of negative factors such as mental illness but also the stimulation of happiness experience and positive skills” (Seligman, 2008). It is acknowledged that the absence of psychopathology (PTH) is not equal to positive mental health (PMH), i.e., high levels of emotional, cognitive, and psychological wellbeing (Keyes et al., 2002). Elements of PMH and PTH are not opposite poles of a single dimension but two negatively correlated factors of mental health (Keyes, 2005).

### The DFM of mental health

“The Dual-Factor Model of Mental Health (DFM)” integrates psychopathology (PTH) and subjective wellbeing (SWB) into a mental health continuum and provides an adjustment and supplement to traditional mental health research paradigms (Greenspoon and Saklofske, 2001; Suldo and Shaffer, 2008; Provencher and Keyes, 2011). Based on the DFM, mental health status may be divided into four categories by measuring subjective wellbeing (SWB) and psychopathology (PTH): complete mental health (high wellbeing, low symptoms of mental illness), symptomatic but content (high wellbeing, high mental illness symptoms), vulnerable (low wellbeing, low mental illness symptoms), and troubled (low wellbeing, high mental illness symptoms) (Doll, 2008; Suldo and Shaffer, 2008). The complete mental health group has the best health condition and develops emotional vitality and good psychological and social functions. Although symptomatic but content people have mental symptoms (such as depression), they also have positive characteristics (such as moderate or high subjective wellbeing). Because of the expansion and formation of positive emotions and positive cognition of life, they are easy to recover from mental illness. Vulnerable people are often overestimated by traditional mental health models and excluded from research and services, because their symptoms of mental diseases do not meet the diagnostic criterion (Suldo and Shaffer, 2008). Troubled ones not only suffer from depression and anxiety but also feel dissatisfied with current life and have poor psychological and social functions. Previous studies have confirmed that the model has a good predictive effect on the physical and mental functions and social function of different groups (Keyes, 2007). Compared with the other three groups, people with complete mental health have better physical condition, psychological function, academic performance, and social function, while troubled people have the worst performance (Renshaw and Cohen, 2014; Antaramian et al., 2015; Wang et al., 2016; Cheng, 2018). It is shown that mental health is not only related to the level of symptoms of mental illness but also to the conscious maintenance of positive psychological quality. Applicability of the DFM with Chinese populations has also been shown to be usable and reliable (Dong et al., 2014; Wang et al., 2016; Xiong et al., 2016; Xiao et al., 2021).

### Self-compassion and mental health

Self-compassion (SC) is a positive self-attitude or emotion regulation strategy and emotional arousal state in which individuals do not dodge their own pain and failure, feel it with an open and tolerant attitude, and give an unbiased understanding (Neff, 2009). Self-compassion plays an active role in mental health, and there is a positive correlation between self-compassion and individual well-being (Neff et al., 2005; Wei et al., 2011; Albertson et al., 2015; Bluth and Blanton, 2015; Greene and Britton, 2015). Self-compassion includes three main components: self-kindness, common humanity, and mindfulness. Self-kindness means that individuals can objectively estimate their own abilities and face their own shortcomings, not being too harsh and critical of themselves, and being more tolerant and understanding themselves. Common humanity means that individuals can realize that pain and disaster are not unique to one person but are experienced by all human beings, and that they are connected and affected by each other rather than isolated individuals. Mindfulness is when an individual can clearly understand their environment, accept it, and try to adjust rather than ignore or magnify their pain. The three elements are closely related to each other. Self-kindness is the emotional component of self-compassion, unconditional acceptance of oneself; common humanity is the cognitive component of self-compassion; it mobilizes the individual's thinking through processes such as social connection and comparison; mindfulness is the advanced adjustment control of self-compassion components, and it optimizes self-kindness and common humanity (Zhang et al., 2010). By activating the individual's self-comfort and self-protection functions, self-compassion reduces the feeling of being threatened, promotes feeling of similar attachment and security, and enhances the sense of well-being (Cheung et al., 2004; Chen et al., 2011; Hu, 2013); the dimensions of self-compassion, self-kindness, and mindfulness have a significant positive correlation with subjective well-being, and there is a significant negative correlation between isolation and subjective wellbeing. There is a significant correlation between self-compassion and total scores on life satisfaction scale and positive and negative emotional scale. People with high self-compassion experience more positive experiences such as optimism and curiosity, and various positive emotions such as excitement, passion, and inspiration. Self-compassion is negatively correlated with depression, anxiety, body shame, and fear of failure (Neff et al., 2005; Odou and Brinker, 2014; Webb et al., 2016). Even after controlling variables such as self-criticism and self-esteem, self-compassion is negatively correlated with anxiety and depression, indicating that people with low levels of self-compassion might be more prone to self-criticism and self-aggression (Gilbert et al., 2010).

### The indirect effect of help-seeking behavior

Help-seeking behavior is an individual coping behavior in the interpersonal field when people suffer from psychological distress (Rickwood et al., 2005). Help-seeking behavior refers to the behavior of actively seeking help from others. They get inner support from other, usually by intimate conversation, is often applied to deal with unpleasant experiences. Generally, the sources of help are divided into two categories: one is from professionals such as professional psychological counselors, and doctors in hospitals, and the other is from non-professionals such as family members, friends or classmates, student tutors, teachers or net friends (Jiang and Wang, 2003). Help-seeking behavior negatively predicts the level of symptoms of mental illness (Qu, 2010; Huang et al., 2020), and professional help-seeking attitude also negatively predicts symptoms of mental illness (Hu, 2011; Bai and Xiao, 2018), indicating that the more the help-seeking behavior, the lower the symptoms of mental illness and that the higher the awareness, willingness, and possibility of help-seeking, the less the symptoms of mental illness. There is a significant positive correlation between self-compassion and professional help-seeking attitude Allen et al., 2012; Heath et al., 2017; Keum et al., 2018; Zhang and Hao, 2019). Students with high levels of self-compassion feel connected, accept each other's opinions easily, and actively help each other when interacting with others, and in general, those with high levels of self-compassion have more social support and positive coping styles and experience less isolation and helplessness (Neff and Beretvas, 2012). Therefore, self-compassion promotes help-seeking behaviors, and those with high levels of self-compassion take more active behaviors to improve their emotions. There is less shame when they fail, less worry about others' judgment when seeking help, less resistance and defense against receiving help and help-seeking behavior, and increase in the probability of seeking help (Allen et al., 2012). Self-compassion also increases self-disclosure by reducing self-stigma when seeking help and increasing help-seeking behavior from professionals (Jiang and Wang, 2003).

### This study

Although the DFM has been widely verified in middle school students and undergraduates (Eklund et al., 2010; Wang et al., 2016; Lai, 2017; Zhang et al., 2018), there are few relevant studies on postgraduates. Despite the postgraduates have relatively high cultural and human capital of the graduate population, with the increase of enrollment and employment pressure, there are also more and more problems such as anxiety, depression and stress. It is necessary to examine the level of mental health and its factors influencing postgraduates. Focusing on positive psychological qualities can help to understand the improvement and solution of the problem.

The aim of this study is to focus on the applicability of the DFM to the mental health status of postgraduates. Based on the research results of undergraduates and the general population, this study assumes that the DFM of mental health is better than the one-factor model. The mental health status of postgraduates will be classified with the DFM quartering method, and the results will be similar to those of the population (Renshaw and Cohen, 2014; Antaramian et al., 2015; Wang et al., 2016; Xiao et al., 2021). In addition, this study also focuses on the construction of positive factors of mental health and intends to find out the relationship between self-compassion and help-seeking behavior and positive factors of mental health. Assumptions are put forward in this study that self-compassion could effectively improve mental health, and that high self-compassion would improve the level of help-seeking, which in turn affects the positive factors of mental health. The state of mental health is often the result of the long-term interaction between individual psychological traits and behavioral responses. Influenced by self-compassion, whether individuals can effectively seek help during stress is an important factor affecting their actual state of mental health. Therefore, it is assumed that help-seeking behavior is an intermediary variable in the relationship between self-compassion and mental health. Because of the positive correlation between self-compassion and positive mental health and the negative correlation between psychological symptoms, this study hypothesizes that the level of self-compassion of postgraduates will affect the two factors of their mental health.

Based on the above research studies, this research hypothesizes:

**Hypothesis 1:** The DFM model could be applied to measure the mental health status of postgraduates, and the model fits better than the one-factor model.
**Hypothesis 2:** Self-compassion is positively correlated with flourishing (positive dimension of mental health) and negatively correlated with symptoms (negative dimension of mental health).
**Hypothesis 3:** Self-compassion positively affects help-seeking behavior.
**Hypothesis 4:** Help-seeking behavior positively affects flourishing (positive dimension of mental health) and negatively affects symptoms (negative dimension of mental health).
**Hypothesis 5:** Help-seeking behavior mediates between self-compassion and mental health.

## Research methods

### Procedures

The random sampling and snowball methods were used to send questionnaire links to postgraduate groups in various departments of certain universities *via* email. All participants gave informed consent to allow their data to be analyzed. The questionnaires were collected from November to December 2020, and a total of 781 questionnaires were collected, including 605 valid questionnaires, with an effective rate of 77.46%.

### Participants

The basic characteristics of the samples are shown in Table 1. The distribution of samples in demographic variables such as gender, major category, and place of origin was relatively balanced, which could reduce the impact of the sample on the research results to a certain extent, meaning that the sample had certain representativeness.

**Table 1:** Basic characteristics of samples (*N* = 605).

**Variable name**		**Sample size**	**Proportion (%)**
Gender	Male	179	29.6
	Female	426	70.4
Grade of postgraduates	First-year	279	46.1
	Second-year	162	26.8
	Third-year or above	164	27.1
Place of origin	Town	360	59.5
	Rural	245	40.5
Major category	Humanities and Arts	170	28.1
	Social Sciences	294	48.6
	Natural Sciences	141	23.3

### Measures

#### Dual-factors of mental health

The Chinese College Students Mental Health Screening Scale (Fang et al., 2018) was used to measure the negative factors of mental health. Internalization symptoms were measured by second-level screening of psychological internalization problems in the College Student Mental Health Screening Scale, with a total of 30 items, including 7 indicators of anxiety, depression, sensitivity, inferiority, social anxiety, somatization, and paranoia. The 7 indicators were scored on a 4-level Likert scale; 1 means “don't think about me at all” and 4 means “very much like me”; the higher the score, the more severe the symptoms or distress. In this study, the Cronbach's a coefficient of the total scale was 0.953, and the subscales were between 0.757 and 0.835, indicating good reliability and validity.

Referring to the research of Xiao et al. (2021), Flourishing Scale (FS) was used to measure the positive factors of mental health more robustly than life satisfaction (Diener et al., 2010). There are 8 items on the flourishing scale, which is scored on a 7-point Likert scale, with “1” representing strongly disagree and “7” representing strongly agree, all of which are scored positively, with a scoring range of 0–56. The scale was based on the self-realization theory and the Flourishing Scale (FS) to re-evaluate the individual's sense of wellbeing; the higher the score, the higher the individual's positive psychological level with more positive psychological resources and social functions. In this study, the internal consistency coefficient of the scale was 0.953, indicating good reliability and validity.

#### Self-compassion

The Chinese version of the Self-Compassion Scale (SCS) was adopted (Chen et al., 2011); the Self-Compassion Scale has a total of 26 items, including the three core components: self-kindness, common humanity, and mindfulness, of which 13 questions were scored positively and 13 questions were scored negatively. The Likert's 5-level scoring method was adopted for all the questions. “1” means never, and “5” means always. This scale was used to measure the level of self-compassion. From the positive and negative aspects, the three core components were further divided into six subdimensions: self-kindness, self-judgment, common humanity, sense of isolation, mindfulness, and over-identification to evaluate the attitude and cognition toward oneself, so as to make the measurement results of self-compassion more comprehensive; the higher the total score, the higher the self-compassion. Numerous research results show that the scale has good reliability and validity, and can stably and effectively measure the level of self-compassion of Chinese college students (Cronbach's a = 0.84). In this study, the Cronbach's a coefficient of the total scale was 0.901, and the subscales were between 0.697 and 0.834, indicating good reliability and validity.

#### Help-seeking behavior

The help-seeking behavior scale mainly refers to the Actual Help-Seeking Questionnaire (AHSQ) (Rickwood and Braithwaite, 1994). This scale is used to measure students' help-seeking behavior, including people whom they seek for help or advice, the frequency of seeking help, and the effectiveness of help they feel when they encounter psychological problems and unpleasant experiences. It includes 2 parts: professional help-seeking and non-professional help-seeking. A total of 10 items are listed. Professional help-seeking includes three items: school therapists, off-campus psychologists, and psychiatrists. Non-professional help includes parents, family members (other than parents), boyfriend/girlfriend, classmates or friends, religious personnel such as priests and monks, teachers or student tutors, net friends, etc. Those who have sought help from the above-mentioned are marked as “1”; otherwise, they are assigned 0 points. The frequency of help-seeking (“1” is rarely, “5” is always) and the level of help (“0” is not helpful, “4” is very helpful) are assessed on a Likert scale of 5 scoring method; First is to find out whether the subject has carried out help-seeking behavior from a certain recourse and second, to measure the frequency of help-seeking behaviors; the higher the frequency, the higher the score, and the more the help-seeking behaviors. Finally, the effectiveness of help the seeker felt was measured, and this item was used as a weighted item to assess the quality of the behavior. Therefore, the score of help-seeking behavior is the product of the three scores under each item, that of professional help is the sum of the scores of the professional help recourses, and that of non-professional help-seeking is the sum of the scores of the non-professional help recourses. Higher scores indicate more help-seeking behavior. In this study, the Cronbach's a coefficient of the total scale was 0.835, and the subscales were between 0.794 and 0.815, indicating good reliability and validity.

### Data analysis

Descriptive statistics and inferential statistics were used to summarize the mental health status of the postgraduates. First of all, the average and standard deviations are used to describe the general situation of postgraduates' mental health, self-compassion, and help-seeking behavior. Second, according to the internalization symptoms and the level of flourishing, four categories of mental health groups are divided into four categories, namely, “complete mental health,” “symptomatic but content,” “vulnerable,” and “troubled mental ill,” and the proportion is described. A structural equation model (SEM) was applied to test which model fits better, the traditional mental health model (one factor model) or the DFM. Then, an ANOVA was carried out to analyze the situations of self-compassion and help-seeking behaviors among the postgraduates. Finally, a linear regression analysis and SEM were used to explore the mediation effect of help-seeking behavior between self-compassion and mental health.

## Results

### Descriptive statistics of the DFM of postgraduate mental health

The results show that the overall mental health status of the postgraduates is good (Table 2). Specifically, in terms of positive psychology, the level of flourishing is relatively high (M = 5.22, SD = 0.99). Referring to previous studies (Lai, 2017), a score of 5 or more indicates a higher level of wellbeing. In terms of negative psychology, the average score of internalization problems is 1.84. In previous studies, a score of more than 2 indicates that there are related psychological problems. It was shown that the level of internalization problems of the postgraduates is generally at a moderate level. Among them, the sensitivity symptom had the highest score (M = 2.23, SD = 0.58), followed by the symptoms of social anxiety, depression, and inferiority, paranoia, anxiety, and somatization. The average score of each dimension was between 1.48 and 2.23.

**Table 2:** Overall mental health of the postgraduates.

**Variable**	**Dimension**	**Factor**	**M**	**SD**	**Md**
Mental health	Flourishing		5.22	0.99	5.25
	Total score of internalization problems		1.84	0.48	1.83
		Anxiety	1.67	0.6	1.50
		Depression	1.89	0.56	2.00
		Sensitive	2.23	0.58	2.25
		Inferiority	1.89	0.56	2.00
		Social anxiety	2.01	0.64	2.00
		Somatization	1.48	0.54	1.25
		Paranoid	1.69	0.55	1.75

M, mean, SD, standard deviation, and Md, mean deviation.

### Test of the DFM of postgraduate mental health

#### Comparison of classification results between one-factor model and DFM

According to the DFM, the score of the flourishing scale represents positive psychology. Referring to previous studies (Lai, 2017), a score of flourishing above 5 points represents high flourishing, while a score below 5 points represents low flourishing. The internalization problem scale is symptom situation. The average score of any symptom of internalization problem ≥ 2 SD is used as the screening standard to classify the level of psychological problems (Liu, 2019). Those with low psychological problems and high flourishing are complete mental health, and those with both high indicators are symptomatic but content. Those with both low indicators represent vulnerable, and those with high psychological problems and low flourishing represent troubled. Under the traditional model, the average score of any symptom of internalization problem ≥ 2 SD represents a person with mental illness; otherwise, it is a person with mental health. The results of the two classification methods on the subject classification are compared, as shown in Table 3.

**Table 3:** Comparison of DFM quartering method and one-factor model classification method.

	**Type**	**Quantity**	**Proportion**	**Flourishing**		**F/t value**	**Internalization** **problems**		**F/t value**
**Division method**				**M**	**SD**		**M**	**SD**	
Quartering method	Completely mental health	383	63.3	5.80	0	338.36^***^	1.66	0	129.82^***^
	Symptomatic but content	15	2.5	5.40	0.43		2.24	0.34	
	Vulnerable	152	25.1	4.23	0.65		1.97	0.39	
	Troubled	55	9.1	3.90	0.66		2.65	0.27	
Traditional method	Mental health	535	88.4	5.35	0.9	9.64^***^	1.75	0.4	−18.74^***^
	Mental illness	70	11.6	4.22	0.88		2.56	0.33	
	Total	605	100%	5.22	0.99		1.84	0.48	

^*^p < 0.05, ^**^p < 0.01, and ^***^p < 0.001.

The DFM was used, and the results showed that in terms of number and proportion, the number of completely mentally healthy people was 383, which was the largest group and accounted for 63.3%; followed by vulnerable people, and the number was 152, accounting for 25.1%. There were 55 people with mental illness (troubled), accounting for 9.1%. The number of symptomatic but content group was 15, which was the smallest group and accounted for 2.5%. From the perspective of flourishing score, completely mentally healthy people (M = 5.8, SD = 0.58) scored the highest, followed by those who were symptomatic but content (M = 5.4, SD = 0.43) and vulnerable (M = 4.23, SD = 0.77), and those who were troubled (M = 3.9, SD = 0.66) scored the lowest (*p* < 0.05). From the point of view of score of internalization, those who were troubled (M = 2.65, SD = 0.27) scored the highest, followed by those who were symptomatic but content (M = 2.24, SD = 0.34) and those who were vulnerable (M = 1.97, SD = 0.39), and those with complete mental health (M = 1.66, SD = 0.38) scored the lowest. An ANOVA was performed on the four groups of people for the problems of agitation and internalization, and it was found that there were significant differences (flourishing dimension F = 338.36, *p* < 0.001; internalization problem dimension F = 129.82, *p* < 0.001), and further *post-hoc* tests found that there were significant differences among the four groups.

According to the traditional one-dimensional model, mental health indicators are mainly psychological symptoms. In this study, there were 535 people with mental health, accounting for 88.4%, and 70 people had mental illness, accounting for 11.6%. An independent sample *t*-test was conducted for the two groups of people on flourishing and internalization problems. It was found that there were significant differences. The flourishing of mentally healthy people was higher than that of those with mental illness (*t* = 9.64, *p* < 0.001), and the score of internalization problems was lower than that of mental illness (*t* = −18.74, *p* < 0.001).

#### Comparison of fitting test of one-factor model and DFM

**Figure 1:**
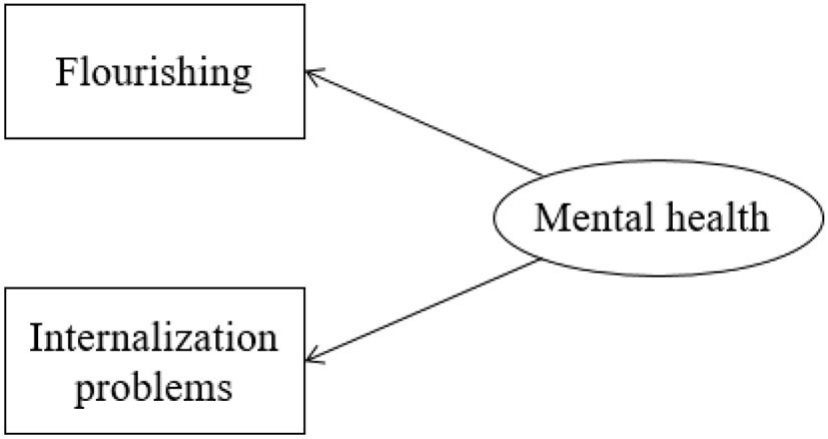
One-factor model of mental health.

**Figure 2:**
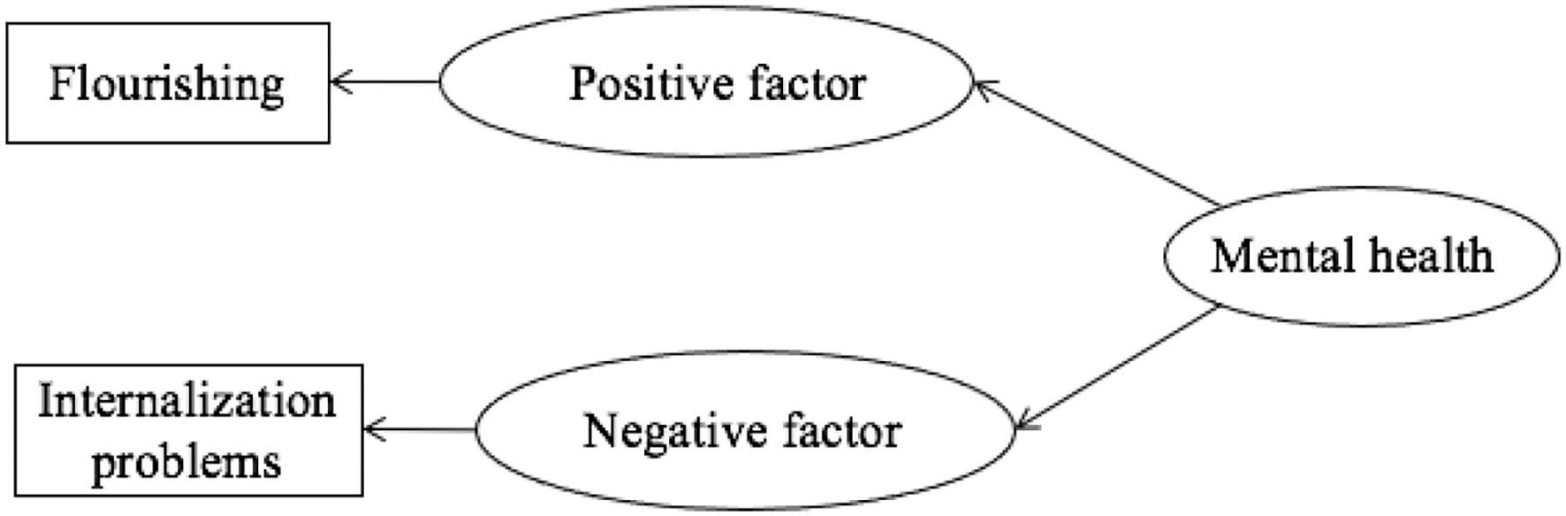
Dual-factor model of mental health.

Referring to the model construction ideas of previous studies (Wang et al., 2016), two test models were constructed, model 1 of mental health one-factor model (as shown in Figure 1) and model 2 of mental health DFM (as shown in Figure 2). Model 1 takes mental health as the only latent variable; items related to flourishing are positive factor loads, and items related to internalization problems are negative factor loads. Model 2 contains two latent variables, positive factor and negative factor. Positive factor corresponds to flourishing projects, while negative factor internalizes problem projects. The model diagram is as follows.

The Amos 22.0 software was used to test the fit degree of the model, and the maximum likelihood method was used to estimate the model. The model fitting path diagram was obtained as follows.

**Figure 3:**
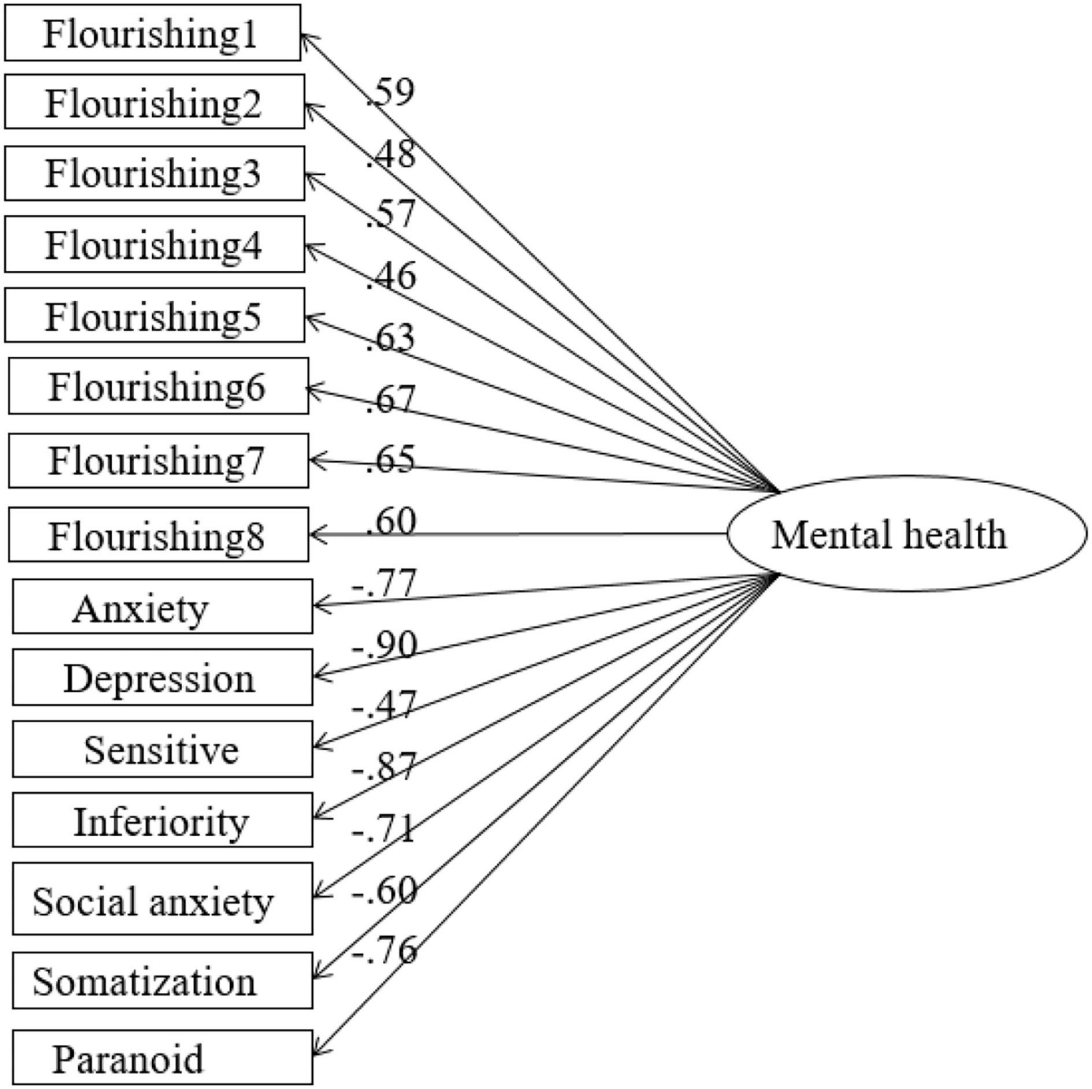
Fitting path data of the one-factor mental health model.

**Figure 4:**
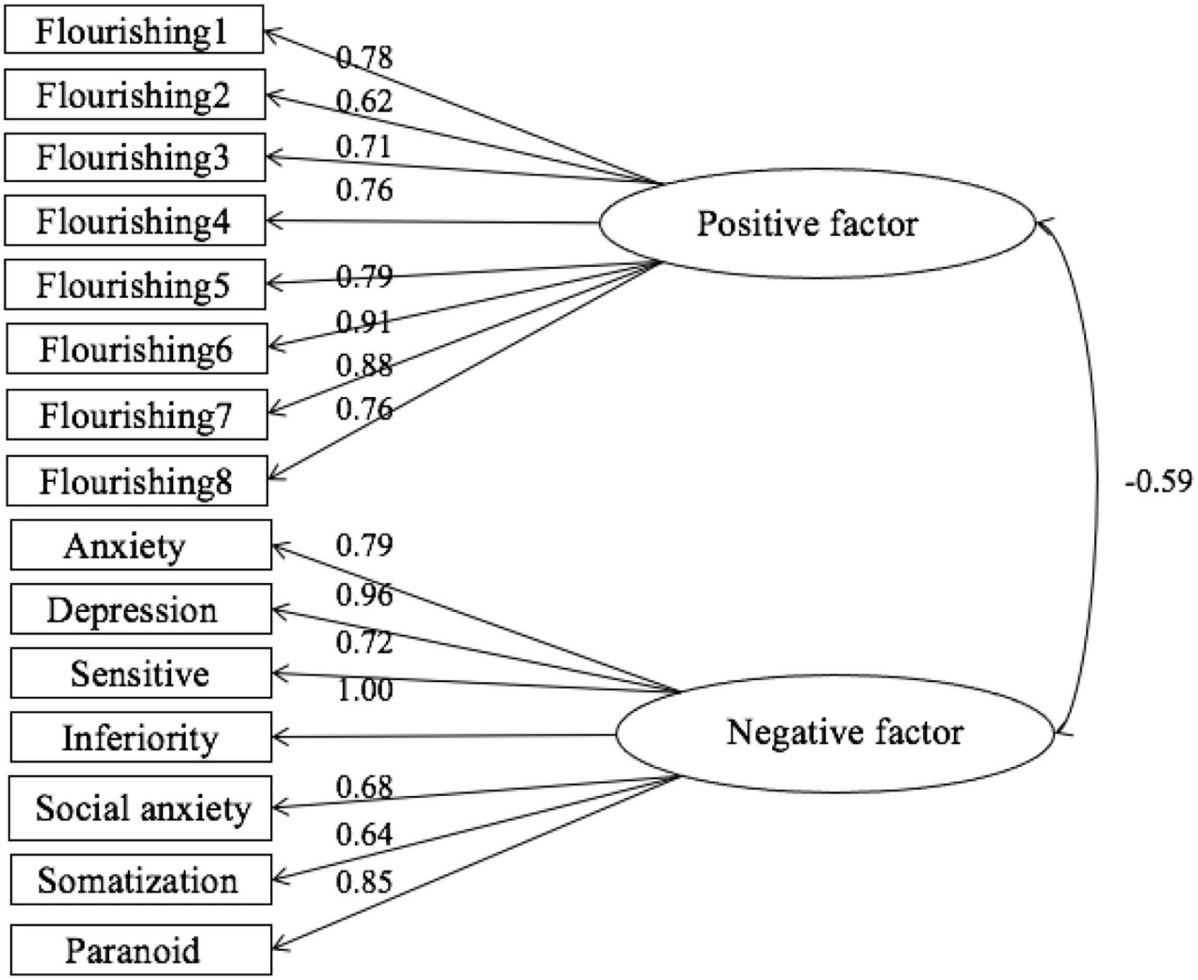
Fitting path data of the dual-factor mental health model.

As shown in Figure 3, the normalized path coefficients of positive factor loads in model 1 ranged from 0.46 to 0.83, and the normalized path coefficients of negative factor loads ranged from 0.6 to 0.9, all of which were significant results (*p* < 0.001). The explanation rate of each factor was between 27 and 81%. As shown in Figure 4, The normalized path coefficients of positive factor in model 2 ranged from 0.62 to 0.91 and were all significant results (*p* < 0.001). The normalized path coefficients of negative factor ranged from 0.64 to 1 and were significant results (*p* < 0.001). The explanation rate of each factor was between 39 and 92%.

As shown in Table 4, the comparison between model 1 (absolute fit index results: χ^2^/df = 18.479, RMSEA = 0.17, GFI = 0.784; relative fit index results: NFI = 0.775, CFI = 0.784) and model 2 (absolute fit index results: χ^2^/df = 2.405, RMSEA = 0.048, GFI = 0.965; relative fit index results: NFI = 0.976, CFI = 0.986) showed that the DFM fits better than the one-factor model.

**Table 4:** Model fitting index.

**Model**	**χ^2^/df**	**GFI**	**CFI**	**NFI**	**RMSEA**
Model 1	18.479	0.666	0.784	0.775	0.17
Model 2	2.405	0.965	0.986	0.976	0.048

3 < χ^2^/df ≤ 5 means acceptable range of model fitting;1 < χ^2^/df ≤ 3 means excellent model fitting; 0.05 < RMSEA ≤0.08 means basic fitting; RMSEA ≤0.05 means high fitting degree; 0.8≤ GFI/NFI/CFI <0.9 means acceptable range; GFI/NFI/CFI ≥0.9 means excellent fitting.

### Self-compassion and help-seeking behavior of postgraduates

#### Self-compassion level of postgraduates

According to the results of this study, the overall self-compassion status of the postgraduates was good (refer to Table 5). Specifically, the mean ranking of the six subdimensions of self-compassion was mindfulness, self-kindness, common humanity, self-judgment, isolation, and over-identification, with scores ranging from 3.02 to 3.51 and all higher than 3 (higher than the median score of 2.5). It was shown that the level of self-compassion of the postgraduates was generally above the middle.

**Table 5:** Overall situation of postgraduates' self-compassion.

**Variable**	**Dimension**	**Factor**	**M**	**SD**
Self-compassion	Overall average	Self-kindness	3.50	0.70
	score (M = 3.33,	Self-judgment	3.29	0.76
	SD = 0.53,	Common humanity	3.43	0.69
	Md = 3.31)	Sense of isolation	3.19	0.84
		Mindfulness	3.51	0.69
		Over identification	3.02	0.80

In order to facilitate the calculation of total scores, self-judgment, isolation, and over-identification were scored in reverse.

#### Help-seeking behavior of postgraduates

In terms of help-seeking behavior, most of the people (75.54%) had sought help from others because of unpleasant experiences in the past 6 months, and only a few (24.46%) had never asked anyone for help. Among the non-professional help-seeking, the top three resources people chose the most were friends or classmates (61.32%), parents (42.64%), family members (except parents) (32.56%). Among the professional help-seeking, the most choice was school psychological counselors (5.79%).

The results are shown in Table 6. The postgraduates had more non-professional help-seeking behavior (M = 18.84, SD = 18.7, but less professional help-seeking behavior (M =0.31, SD = 1.61). It was shown that the postgraduates were more inclined to seek help from non-professionals and less from professionals when encountering psychological problems.

**Table 6:** Scores of help-seeking behavior of the postgraduates in the past 6 months.

**Variable**	**Dimension**	**Factor**	**M**	**SD**	**Md**
Help-seeking behavior	Help-seeking behavior (M = 19.15, SD = 18.9,Md = 15)	Non-professional help-seeking behavior	18.84	18.7	15
		Professional help-seeking behavior	0.31	1.61	0

According to the three indexes related to help-seeking behavior, namely, whether to ask for help from the others, the frequency of asking for help, and the effectiveness of the help, the table adds up the product of the three scores under each item to obtain 2 factors of non-professional help-seeking behavior and professional help-seeking behavior.

### Hypothesis testing

#### The relationship between self-compassion and mental health

It is shown in Table 7 that there is a significant correlation between self-compassion and mental health. There was a significant positive correlation between self-compassion and its dimensions and flourishing (*p* < 0.01), and it was significantly negatively correlated with the total score of internalization problems and the seven factors of anxiety, depression, sensitive, inferiority, social anxiety, somatization, and paranoia (*p* < 0.01).

**Table 7:** Correlation between self-compassion and mental health.

	**Flourishing**	**Total score of internalization problems**	**Anxiety**	**Depression**	**Sensitive**	**Inferiority**	**Social anxiety**	**Somatization**	**Paranoid**
Self-compassion	0.505^**^	−0.68^**^	−0.549^**^	−0.648^**^	−0.589^**^	−0.634^**^	−0.535^**^	−0.415^**^	−0.533^**^
Self-kindness	0.296^**^	−0.326^**^	−0.243^**^	−0.326^**^	−0.255^**^	−0.315^**^	−0.281^**^	−0.185^**^	−0.258^**^
Common humanity	0.233^**^	−0.234^**^	−0.209^**^	−0.226^**^	−0.178^**^	−0.214^**^	−0.174^**^	−0.157^**^	−0.189^**^
Mindfulness	0.287^**^	−0.333^**^	−0.276^**^	−0.317^**^	−0.263^**^	−0.294^**^	−0.287^**^	−0.219^**^	−0.259^**^
Self-judgment	0.414^**^	−0.594^**^	−0.474^**^	−0.57^**^	−0.517^**^	−0.562^**^	−0.464^**^	−0.363^**^	−0.461^**^
Sense of isolation	0.441^**^	−0.67^**^	−0.533^**^	−0.628^**^	−0.576^**^	−0.641^**^	−0.522^**^	−0.419^**^	−0.53^**^
Over identification	0.425^**^	−0.645^**^	−0.535^**^	−0.606^**^	−0.636^**^	−0.583^**^	−0.478^**^	−0.373^**^	−0.499^**^

^*^p < 0.05, ^**^p < 0.01, and ^***^p < 0.001.

#### The relationship between self-compassion and help-seeking behavior

It is shown in Table 8 that self-compassion and all the dimensions except for over-identification are positively correlated with the total score of help-seeking (*p* < 0.05), among which professional help-seeking behavior was not significantly related to self-compassion and each dimension. Self-compassion and all the dimensions except over-identification were positively correlated with non-professional help-seeking behavior (*p* < 0.05).

**Table 8:** Correlation between self-compassion and help-seeking behavior.

	**Total score of help-seeking behavior**	**Professional help-seeking behavior**	**Non-professional help-seeking behavior**
Total score of self-compassion	0.134^**^	−0.070	0.141^**^
Self-kindness	0.149^**^	−0.039	0.154^**^
Common humanity	0.161^**^	−0.049	0.167^**^
Mindfulness	0.113^**^	−0.038	0.118^**^
Self-judgment	0.076^*^	−0.058	0.082^*^
Sense of isolation	0.098^*^	−0.037	0.102^*^
Over identification	−0.025	−0.073	−0.019

^*^p < 0.05, ^**^p < 0.01, and ^***^p < 0.001.

#### The relationship between help-seeking behavior and mental health

It is shown in Table 9 that there is a significant positive correlation between the total score of help-seeking behavior and non-professional help-seeking behavior and flourishing (*p* < 0.01). There was a significant negative correlation between the total score of help-seeking behavior and the total score of internalization problems, social anxiety, and paranoia (*p* < 0.05). There was a significant positive correlation between professional help-seeking behavior and the total score of internalization problems, anxiety, and paranoia (*p* < 0.05). There was a significant negative correlation between non-professional help-seeking behavior and the total score of internalization problems, depression, inferiority, social anxiety, and paranoia (*p* < 0.05).

**Table 9:** Correlation between help-seeking behavior and mental health.

	**Flourishing**	**Total score of internalization problems**	**Anxiety**	**Depression**	**Sensitive**	**Inferiority**	**Social anxiety**	**Somatization**	**Paranoid**
Total score of help-seeking behavior	0.209^**^	−0.095^*^	−0.049	−0.102^*^	−0.020	−0.078	−0.118^**^	−0.069	−0.110^**^
Non-professional help-seeking behavior	−0.073	0.088^*^	0.118^**^	0.076	0.075	0.073	0.017	0.063	0.090^*^
Professional help-seeking behavior	0.217^**^	−0.104^*^	−0.060	−0.110^**^	−0.026	−0.085^*^	−0.120^**^	−0.075	−0.119^*^

^*^p < 0.05, ^**^p < 0.01, and ^***^p < 0.001.

#### Regression analysis of self-compassion, help-seeking behavior, and mental health of postgraduates

A linear regression analysis was carried out to set up the model by taking flourishing as the dependent variable and self-compassion and help-seeking behavior as independent variables. The results showed that the multivariate correlation coefficient R was 0.534, the coefficient of determination R^2^ was 0.295, and the adjusted coefficient of determination ΔR^2^ was 0.285 (Table 10). The analysis of variance showed that the results are significant (Table 11). The influence of the three variables of non-professional help-seeking behavior as predictors on the dependent variable reached a significant level, and the dimension of mindfulness and self-judgment (*p* = 0.056; *p* = 0.062) reached a marginally significant level.

**Table 10:** Summary of the flourishing model.

**Model**	**R**	**R^2^**	**ΔR^2^**	**F**
1	0.543	0.295	0.285	31.153^***^

^*^p < 0.05, ^**^p < 0.01, and ^***^p < 0.001.

**Table 11:** Coefficient of the flourishing regression model.

	**Non-standard partial regression coefficient**		**Standard partial regression coefficient**	**t**
	**B**	**Standard error**	**Beta**	
Self-kindness	0.064	0.083	0.045	0.765
Common humanity	0.084	0.071	0.059	1.189
Mindfulness	0.154	0.081	0.108	1.913
Self-judgment	0.142	0.076	0.109	1.872
Sense of isolation	0.194	0.068	0.165	2.874^**^
Over identification	0.229	0.075	0.185	3.063^**^
Professional help-seeking behavior	−0.032	0.021	−0.052	−1.508
Non-professional help-seeking behavior	0.009	0.002	0.170	4.760^***^

^*^p < 0.05, ^**^p < 0.01, and ^***^p < 0.001.

The total score of internalization problems was taken as the dependent variable. Self-compassion (self-kindness, common humanity, mindfulness, self-judgment, isolation, and over-identification), help-seeking behavior (non-professional help-seeking behavior and professional help-seeking behavior) were used as independent variables; the input regression analysis was carried out using the method, and the results showed that the multivariate correlation coefficient R was 0.736, the coefficient of determination R^2^ was 0.641, and the adjusted coefficient of determination ΔR^2^ was 0.535 (Table 12). The variance analysis showed that the results are significant (Table 13); isolation and over-identification, as predictors, had a significant impact on the dependent variable.

**Table 12:** Summary of the internalization problem model.

**Model**	**R**	**R^2^**	**ΔR^2^**	**F**
1	0.736	0.641	0.535	87.82^***^

^*^p < 0.05, ^**^p < 0.01, and ^***^p < 0.001.

**Table 13:** Model coefficients of internalization problems.

	**Nonstandard partial regression coefficient**		**Standard partial regression coefficient**	**t**
	**B**	**Standard error**	**Bate**	
Self-kindness	−0.01	0.032	−0.015	−0.320
Common humanity	−0.035	0.027	−0.050	−1.265
Mindfulness	−0.1	0.031	−0.146	−3.204^***^
Self-judgment	−0.079	0.029	−0.126	−2.685^**^
Sense of isolation	−0.194	0.026	−0.344	−7.415^***^
Over identification	−0.151	0.029	−0.253	−5.203^***^
Professional help-seeking behavior	0.013	0.008	0.044	1.570
Non-professional help-seeking behavior	−0.001	0.001	−0.039	−1.342

^*^p < 0.05, ^**^p < 0.01, and ^***^p < 0.001.

#### Mediating analysis of self-compassion, help-seeking behavior, and mental health of postgraduates

Referring to the results of the above multiple regression analysis, insignificant paths were deleted, and a structural equation model was constructed to test the mediating effect of self-compassion, help-seeking behavior, and mental health. The settings were as follows: self-judgment, isolation, mindfulness, and over-identification constituted self-compassion. Non-professional help-seeking behavior was used as a mediating variable and flourishing as a dependent variable were all significant. The final model includes 6 explicit variables and 1 latent variable.

Using AMOS22.0 for model fitting analysis, the results are as follows:

The research results (Table 14) show that the fitting degree of the mediation model reaches the standard. Specifically, the results of the mediation model (absolute fit index: χ^2^/df = 1.6, RMSEA = 0.032, GFI = 0.995; relative fit index: NFI = 0.993, CFI = 0.997) was excellent, and all the indicators were well-fitted, indicating that the mediation model is acceptable. The output of the model is as follows.

**Table 14:** Fitting index of the mediation model.

**Model**	**χ^2^/df**	**GFI**	**CFI**	**NFI**	**RMSEA**
Mediation model	1.600	0.995	0.997	0.993	0.032

**Figure 5:**
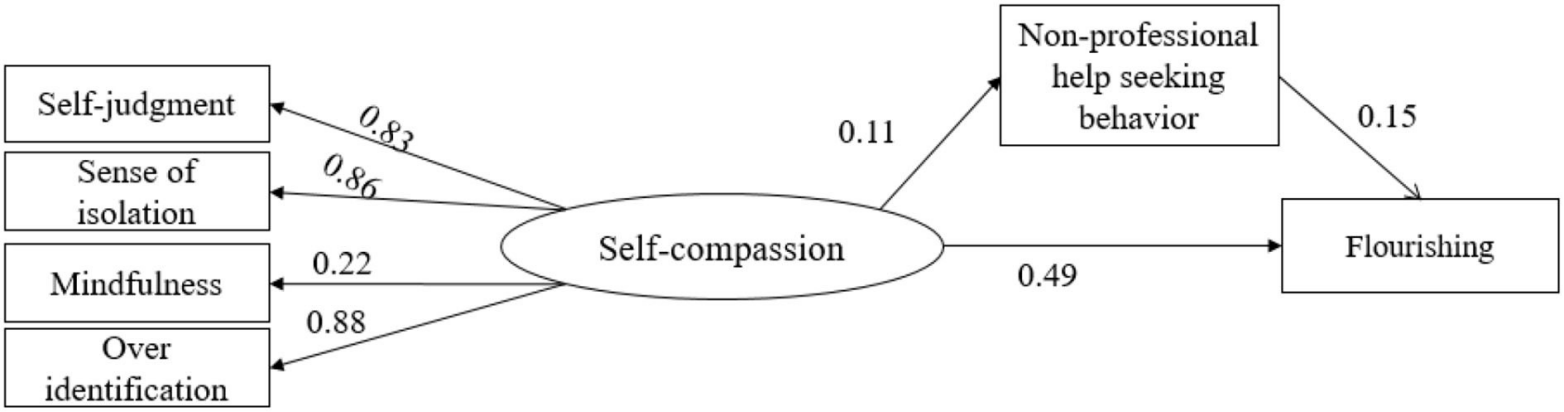
Mediating model of non-professional help-seeking behavior between self-compassion and flourishing.

The mediating effect of the model was tested with the Bias-Corrected Bootstrap procedure. Using the repeated random sampling technique to draw 2,000 bootstrap samples from the original data, an approximate sampling distribution was generated, and a 95% confidence interval model for the mediation effect was estimated using the 2.5th and 97.5th percentiles. The results of the data verified that non-help-seeking behavior played a mediating role in the prediction of self-compassion and flourishing, as shown in Figure 5. In this model, the size of the direct effect of self-compassion on flourishing was 0.49, *p* < 0.001, and the size of the mediating effect was 0.11^*^0.15 = 0.0165 (*p* < 0.001).

## Discussion

This study found that the DFM is applicable for measurement of the mental health of the postgraduates. Combined with the model path coefficient and interpretation rate results, it was shown that the DFM fit better than the traditional one-dimensional mental health model, and that the DFM could be applied to the tested sample group and was more suitable for explaining the mental health status of the postgraduates, which supports hypothesis 1. The DFM of mental health was better than the one-factor model, which confirmed the rationality of evaluating mental health from two dimensions of positive factor and negative factor. It shows that the traditional way of defining mental health as the absence of mental illness has the potential to ignore the positive factors of mental health and underestimate people's resilience. According to the DFM, the postgraduates can be divided into complete mental health (63.3%), symptomatic but content (2.5%), vulnerable (25.1%), and troubled (9.1%). In general, the postgraduates had a good level of mental health and less internalized psychological problems. The internalized psychological problems were mainly sensitivity, social anxiety, depression, and inferiority. The proportion of the four groups in this study was basically consistent with previous research results (Eklund et al., 2010; Wang et al., 2016; Lai, 2017; Zhang et al., 2018). It was found that the proportion of people with complete mental health was the highest, and that the proportion of people with symptomatic but content was the lowest. However, compared with other studies, the proportion of people with complete mental health and those who were vulnerable in this study was relatively high, and the proportion of people who were symptomatic but content and those who were troubled was relatively low. The reason might be that the postgraduates enjoyed more social resources and school environmental protection, and that they had better human and social resources and supports. Therefore, the proportion of completely mental health was higher than other groups, and there was fewer troubled (Zhang et al., 2018). Due to the competitive pressure and high expectation brought by their own identity advantages, they also faced high academic challenges, which could easily lead to their feeling of pressure and reduced happiness (Lu et al., 2008). Therefore, although they had mild psychological symptoms, they lacked a positive psychological experience. At present, mental health management in colleges and universities is still mainly focused on students with serious psychological problems (Jiang and Wang, 2003). There is a lack of effective support and intervention measures for individuals with mild psychological disorders and susceptible groups. In addition, students rarely seek professional help to solve their psychological problems (Fang et al., 2018). Therefore, they also lack mental health care resources. The symptomatic but content group that could coexist with mental disorders and has a better psychological experience accounts for the least proportion.

The symptomatic but content and vulnerable groups could not be identified by traditional models, but they have different characteristics and need more attention. The symptomatic but content group has high levels of mental health, so despite the symptoms of mental illness, they can actively participate in learning and social life, and are more likely to repair themselves and develop in a positive way of living (Gokcen et al., 2012). Therefore, they should be encouraged to develop their own inner strength and understand their self-relationship with symptoms. On the contrary, for the vulnerable group, although they show PTH, because of their low flourishing, they are more likely to be converted into troubled when they encounter troubles or pressures. Vulnerable groups are often ignored by the psychological appraisement system based on the traditional model (Fang et al., 2018) and do not get guidance and help on time (Jiang and Wang, 2003). With the increasing competition and psychological symptoms among postgraduates, it is very important to cultivate the abilities of self-compassion and help-seeking behavior.

In this study, the highest levels of internalization problems of the postgraduate students are sensitivity and social anxiety, which is somewhat different from previous studies focusing on depression or anxiety (Hu, 2013; Yang et al., 2015; Xiao et al., 2021). The reason is that for most of the postgraduates, the emotional distress in their daily life is not severe enough to be depressive or anxious, and the main distress comes from interpersonal sensitivity and social distress, especially if the relationship with the tutor may be an important indicator of the mental health level of the postgraduates (Zhang et al., 2018). At the same time, there may also be individuals who lack awareness and introspection about psychological problems and tend to attribute problems to external causes such as interpersonal tension (Wang et al., 2015).

Consistent with existing results (Yang et al., 2015), this study found that the overall level of the postgraduates' self-compassion was relatively good, that the overall difference was small, and that most of them were at the upper-middle level. Self-compassion is significantly positively correlated with the positive dimension of mental health and significantly negatively correlated with the negative dimension of mental health, which supports hypothesis 2. In general, the postgraduates have better self-awareness ability and could have a certain understanding and control of their own cognitive and emotional processes (Zhang et al., 2018). When encountering setbacks or difficulties, they could give themselves more understanding and tolerance, view their own shortcomings correctly, evaluate and understand themselves reasonably, reduce self-attacks and negative judgments on themselves, and believe that they are connected with others. They are not isolated individuals and could make efforts to get themselves out of difficulties rather than get blindly immersed in negative cognitions or emotions (Lu et al., 2008). Self-compassion could positively predict the positive mental health of postgraduates and negatively predict internalized psychological problems (Chen et al., 2011).

This study also found that the postgraduates had a high incidence of help-seeking behavior, and that more than 70% of them chose to ask others for help when they encountered psychological distress. Consistent with the results of previous studies (Gu, 2013; Hu, 2013; Li, 2013), it was found that there was a positive correlation between self-compassion and help-seeking behavior, and that the correlation was mainly focused on non-professional help-seeking, which partially supports hypothesis 3 and indicates that the students' self-compassion-oriented help-seeking behavior was mainly targeted at non-professional social networks around them such as friends, parents, and family rather than choosing professionals. This is consistent with the results of previous studies on college students (Jiang and Wang, 2003; Wang et al., 2015). The reasons for this result might be as follows: first, it might be related to Chinese cultural atmosphere that talking about personal emotions will be regarded as revealing personal vulnerability and that family troubles should not be talked about in public, leading to negative attitudes toward seeking professional help (Guo, 2015). When people feel that the society has higher tolerance for professional help-seeking behavior, they are more inclined to seek help (Xia, 2005). Second, it is related to the accessibility and trust of professionals. People of non-professional help are usually from one's social network. They are more connected with students' daily life, more familiar, more trustworthy, more accessible, and more convenient. On the contrary, school counselors might be hindered by the multiple identities of college student tutors (Qin and Gao, 2012). Postgraduates may not recognize the effect of professional, lack of trust and are unwilling to seek professional help.

Help-seeking behavior is significantly positively correlated with positive factors of mental health and significantly negatively correlated with negative factors of mental health (total score of internalization problems and depression, social anxiety, and paranoia). Professional help-seeking is significantly correlated with positive factors of mental health, and it was significantly negatively correlated with negative mental health factors (total score of internalization problems and depression, inferiority, social anxiety, and paranoia). Non-professional help-seeking is significantly positively correlated with negative factors of mental health (total score of internalization problems and social anxiety and paranoia), which basically supports hypothesis 4. This is consistent with the results of previous studies on college students (Bai and Xiao, 2018; Keum et al., 2018). Help-seeking as a positive coping style was negatively correlated with all factors of SCL-90 (Lu et al., 2008) among postgraduates. Subjective wellbeing is positively correlated with help-seeking behavior in a positive coping style (Shi et al., 2011; Wang, 2017). College students with positive help-seeking qualities have a healthier mental state, and college students who are more willingly to ask for help are more likely to maintain a positive mood, enhance their subjective feelings of happiness and joyfulness, and improve their mental health (Nam et al., 2013).

The mediating effect of this study showed that non-professional help-seeking behavior played a partial mediating role in self-compassion and positive mental health, which partially supported for hypothesis 5. It can be seen that self-compassion has made a major contribution to positive mental health (Allen et al., 2012), indicating that self-compassion is an important influence for postgraduates to maintain mental health. On the one hand, self-compassion could directly affect positive mental health. People with self-compassion are more tolerant to themselves and have a stronger sense of connection with others (Bluth and Blanton, 2015). They could use some strategies to help themselves out of a predicament, which could improve their positive experience (Albertson et al., 2015). This also explains the influence of self-compassion on help-seeking behavior. People who actively engage in self-compassion improve their emotional awareness and cognitively and emotionally accept the fact that they need help and make the behavior of seeking help from others (Gu, 2013). Help-seeking behavior requires individuals to rely on the strength of others, and it requires certain social relations and communication skills (Rickwood et al., 2005). Self-compassion has a positive effect on improving personal relationships and trusting others (Crocker et al., 2010). On the other hand, self-compassion could affect positive mental health by influencing the behavior of obtaining help from social networks such as friends and family, which shows that caring for oneself is a positive self-attitude (Guo, 2015). Help seeking behavior requires individuals to rely on the strength of others, it requires certain social relations and communication skills (Rickwood et al., 2005). Self-compassion could promote individuals to seek help by communicating with others. It was shown that the improvement of self-compassion and the behavior of being good at asking for help could effectively improve the positive psychological quality of individuals (Shi et al., 2011).

## Limitations and future prospects

There are still limitations on the current study. First, the sampling source has certain limitations, and the sample size is relatively small; contemplation of geographic area and courses is relatively insufficient, so it is difficult to generalize the results. Second, with convenience sampling, there may be selection bias and potential threats. Finally, this study only involves the results of one survey and lacks long-term and longitudinal tracking of the development trend of individual mental health and changes with the development of academic study as well as the dynamic understanding of the two factors of mental health and the intervention of negative factors.

Therefore, as part of future research, follow-up research should be designed and implemented using multiple data collection methods. Longitudinal research should be carried out after conducting a questionnaire survey. Future research should include longitudinal data or experimental methods to verify their relationships, extend to other types of master's degrees and other geographical realities, and analyze in a factorial way to better cover the universe studied.

## Conclusion and value

By DFM measurement of postgraduates' mental health, it was found that the overall mental health of the postgraduates was good, and that the main internalized problems include sensitivity, social anxiety, depression, and inferiority. Self-compassion significantly positively predicted positive factors of mental health, and significantly negatively predicted negative factors of mental health. The postgraduates mainly used non-professional help-seeking methods to seek outside help, which positively predicted the positive factors of mental health. Non-professional help-seeking played a partial mediating role between self-compassion and positive factors of mental health.

This study verifies the applicability of the DFM in the mental health assessment of postgraduates, which not only enriches the applicable groups of DFM but also provides a more comprehensive and accurate method for evaluating the mental health of postgraduates. It was also found that the postgraduates had a high level of self-compassion and help-seeking behavior, which had a strong correlation with the overall mental health and social adaptation of the postgraduate group. Moreover, it was found that non-professional help-seeking played a partial mediating role between self-compassion and positive factor of mental health, suggesting that further mental health education needs to prompt the individual's ability of self-care and the awareness of active help seeking, which can effectively improve the positive factor of mental health, thereby improving the individual's mental health and social adaptability. It may be necessary to build a more appropriate general education for students on how to maintain mental health and conduct self-compassion to help individuals acquire knowledge and learn skills, develop grit and optimistic attitude, and learn to keep their mind and body balanced.

## Data availability statement

The raw data supporting the conclusions of this article will be made available by the authors, without undue reservation.

## Author contributions

LMi designed the study. LMi and LMe analyzed the data. LMi and NJ wrote and modified the manuscript. All authors have read and agreed to the published version of the manuscript.

## Funding

This work was funded by the 2021 Fujian Social Science Planning Project (FJ2021B211).

## Conflict of interest

The authors declare that the research was conducted in the absence of any commercial or financial relationships that could be construed as a potential conflict of interest.

## Publisher's note

All claims expressed in this article are solely those of the authors and do not necessarily represent those of their affiliated organizations, or those of the publisher, the editors and the reviewers. Any product that may be evaluated in this article, or claim that may be made by its manufacturer, is not guaranteed or endorsed by the publisher.
